# Prognostic value of serum CRP, IgM and IgA levels in children with Mycoplasma pneumoniae and pleural effusion

**DOI:** 10.5937/jomb0-54947

**Published:** 2025-09-05

**Authors:** Huiqing Guo

**Affiliations:** 1 Children's Hospital of Shanxi and Women's Health Center of Shanxi, Department of Pediatrics, Taiyuan City, Shanxi Province, China

**Keywords:** mycoplasma pneumoniae pneumonia, pleural effusion, C-reactive protein, Immunoglobulin M, Immunoglobulin A, pediatric pneumonia, biomarker, upala pluća koju izaziva mycoplasma pneumoniae, pleuralni izliv, C-reaktivni protein, imunoglobulin M, imunoglobulin A, pedijatrijska pneumonija, biomarkeri

## Abstract

**Background:**

Mycoplasma pneumoniae pneumonia (MPP) is a common cause of community-acquired pneumonia in children, with pleural effusion (PE) as a recognised but challenging complication. Identifying reliable biomarkers to predict PE in MPP is crucial for timely intervention. This study aims to evaluate the prognostic value of serum C-reactive protein (CRP), Immunoglobulin M (IgM), and Immunoglobulin A (IgA) levels in children with MPP and PE.

**Methods:**

A retrospective study was conducted on 200 pediatric patients diagnosed with MPP between January 2021 and December 2023. Patients were divided into two groups: MPP with PE (n=100) and MPP without PE (n=100). Serum CRP , IgM, and IgA levels were measured, and their associations with PE were analysed using logistic regression models.

**Results:**

The MPP with PE group had significantly higher CRP (30.22±24.01 mg/L vs 9.90±7.01 mg/L, P<0.001) and IgM (167.39±85.68 mg/dL vs 130.48±77.65 mg/dL, P=0.002) levels compared to the MPP without PE group. In contrast, IgA levels were significantly lower in the PE group (164.50±87.22 mg/dL vs. 195.51±79.93 mg/dL, P=0.009). Multivariate logistic regression analysis revealed that elevated CRP (OR=1.255, 95% CI: 1.132-1.391, P<0.001) and IgM (OR=1.795, 95% CI: 1.777-4.867, P=0.001) were independent risk factors for PE, while higher IgA levels were protective (OR=0.281, 95% CI: 0.131-0.602, P=0.001).

**Conclusions:**

Serum CRP and IgM levels are potential predictors of pleural effusion in children with MPP , while elevated IgA levels may indicate a lower risk. These biomarkers could aid in early risk stratification and guide clinical management to improve outcomes.

## Abstract (sr)

**Uvod:**

Mycoplasma pneumonia (MPP) predstavlja čest uzrok zapaljenja pluća u zajednici kod dece, pri čemu se javlja pleuralni izliv (PE) što je komplikacija koja nije jednostavna. Identifikovanje pouzdanih biomarkera za predviđanje PE kod MPP od suštinskog je značaja za pravovremenu intervenciju. Cilj ove studije je bio da se proceni prognostička vrednost nivoa serumskog C-reaktivnog proteina (CRP), imunoglobulina M (IgM) i imunoglobulina A (IgA) kod dece sa MPP i PE.

**Metode:**

Sprovedena je retrospektivna studija na 200 pediatrijskih pacijenata kod kojih je dijagnostifikovana MPP u periodu od januara 2021. do decembra 2023. godine. Pacijenti su podeljeni u dve grupe: MPP sa PE (n=100) i MPP bez PE (n=100). Izmereni su nivoi serumskog CRP-a, IgM-a i IgA, a njihova povezanost sa PE analizirana je korišćenjem logističkih regresionih modela.

**Rezultati:**

Grupa sa MPP i PE je imala značajno više vrednosti CRP-a (30,22±24,01 mg/L naspram 9,90±7,01 mg/L, P<0,001) i IgM-a (167,39±85,68 mg/dL naspram 130,48±77,65 mg/dL, P=0,002) u poređenju sa grupom bez PE. Nasuprot tome, nivoi IgA su bili značajno niži u grupi sa PE (164,50±87,22 mg/dL naspram 195,51±79,93 mg/dL, P=0,009). Multivarijantna logistička regresiona analiza je pokazala da su povišeni CRP (OR=1,255, 95% CI: 1,132-1,391, P<0,001) i IgM (OR=1,795, 95% CI: 1,777-4,867, P=0,001) nezavisni faktori rizika za PE, dok su viši nivoi IgA pokazali zaštitni efekat (OR=0,281, 95% CI: 0,131-0,602, P=0,001).

**Zaključak:**

Nivoi serumskog CRP-a i IgM-a mogu da predstavljaju potencijalne prediktore pojave pleuralnog izliva kod dece sa MPP , dok povišeni nivoi IgA mogu ukazivati na manji rizik. Ovi biomarkeri mogu da doprinesu ranoj stratifikaciji rizika i usmeravanju kliničkih aktivnosti radi poboljšanja ishoda.

## Introduction

Mycoplasma pneumoniae (M. pneumoniae) is a common bacterial pathogen that causes respiratory infections, particularly in children. It is a leading cause of community-acquired pneumonia (CAP) in pediatric populations, especially among children aged 5 to 15 years. The pathogen is unique in that it lacks a cell wall, making it resistant to beta-lactam antibiotics, and its clinical manifestations can vary widely, ranging from mild upper respiratory tract infections to more severe pneumonia [1]. While most cases of M. pneumoniae pneumonia (MPP) are self-limiting and respond to appropriate antibiotic therapy, some children experience complications that necessitate closer monitoring and more aggressive treatment. One such complication is pleural effusion (PE), which occurs when fluid accumulates in the pleural cavity, often as a result of infection or inflammation.

Pleural effusion associated with M. pneumoniae pneumonia is a well-recognised but complex complication. It can manifest as a localised or parapneumonic effusion, often accompanied by an inflammatory response that can be difficult to manage. Children with M. pneumoniae pneumonia complicated by pleural effusion may experience prolonged hospitalisations and, in severe cases, require surgical intervention such as thoracentesis or chest tube drainage [2]. The pathophysiology of pleural effusion in these patients involves the bacterial invasion and inflammation of the pleural lining, which leads to an imbalance in the normal fluid exchange process. This inflammation can further exacerbate respiratory distress and complicate the recovery process. Thus, early recognition and effective management of pleural effusion are critical to improving clinical outcomes for these children [3].

Diagnosing and predicting the development of pleural effusion in children with M. pneumoniae infection remains challenging. Traditional clinical assessments, while valuable, often lack the sensitivity and specificity needed to predict complications like pleural effusion accurately. Thus, there is growing interest in identifying reliable biomarkers that could assist in the early identification of children at high risk for severe outcomes, including pleural effusion [4]. Serum biomarkers, in particular, have been studied as potential prognostic tools in respiratory infections, as they may reflect the intensity of inflammation and the body’s immune response. Among the biomarkers under investigation, C-reactive protein (CRP), Immunoglobulin M (IgM), and Immunoglobulin A (IgA) have shown promise.

C-reactive protein (CRP) is an acute-phase reactant produced by the liver in response to inflammation. It is widely used as a marker of systemic inflammation and infection [5]. In the context of M. pneumoniae infection, CRP levels are typically elevated, reflecting the degree of inflammatory response to the bacterial pathogen. Several studies have demonstrated that high CRP levels correlate with the severity of pneumonia and can serve as an indicator of complications such as pleural effusion [6]. Elevated CRP levels have been associated with more severe disease courses and poorer outcomes in children with M. pneumoniae pneumonia. Thus, CRP may provide valuable prognostic information for clinicians managing these patients.

Immunoglobulin M (IgM) is the first antibody produced in response to an infection and is a critical component of the body’s early immune response. In M. pneumoniae infections, the presence of IgM is commonly used as a diagnostic marker, as it typically appears early in the course of the illness [7]. Elevated IgM levels have been linked to the acute phase of infection and are often used to confirm recent exposure to the pathogen. Moreover, studies have suggested that higher IgM levels may be associated with more severe manifestations of M. pneumoniae pneumonia, including the development of pleural effusion [8]. Therefore, IgM may serve not only as a diagnostic marker but also as a potential prognostic tool in identifying children at risk for complications.

Immunoglobulin A (IgA), primarily found in mucosal secretions, plays a crucial role in the immune defence of the respiratory tract. It is an important part of the local immune response and protects against respiratory pathogens such as M. pneumoniae [9]. While IgA levels in the serum are typically lower than those of IgM, they may be elevated in response to mucosal infections. In the case of M. pneumoniae pneumonia, IgA production may increase as the immune system attempts to control the disease locally. Some studies suggest that elevated serum IgA levels may indicate a more intense or prolonged infection, which could increase the likelihood of complications like pleural effusion [10].

The relationship between serum CRP, IgM, and IgA levels and the development of pleural effusion in children with M. pneumoniae pneumonia is still an area of ongoing research. Although several studies have explored the role of these biomarkers in predicting disease severity, there is a lack of consensus regarding their precise prognostic value, particularly in the context of pleural effusion [11] [12]. The challenge lies in understanding how these markers interact with the clinical characteristics of M. pneumoniae infection and how they can be used to guide early interventions. Understanding these dynamics could significantly enhance the ability to predict which children are at high risk for developing pleural effusion, allowing for earlier and more targeted treatment.

This study aims to explore the prognostic value of serum CRP, IgM, and IgA levels in children with M. pneumoniae pneumonia complicated by pleural effusion. By analysing the association between these biomarkers and the development of pleural effusion, this research seeks to provide valuable points into their potential role as predictive tools. The findings of this study could aid clinicians in identifying high-risk patients early in the disease course, allowing for timely interventions that may improve patient outcomes. Moreover, this research could help refine the clinical management of pediatric patients with M. pneumoniae pneumonia, leading to better-targeted therapies and a reduction in complications such as pleural effusion.

## Materials and methods

### General information

This retrospective study focused on children diagnosed with Mycoplasma pneumoniae pneumonia (MPP) in the pediatric ward of our hospital between January 2021 and December 2023. A total of 200 children were included in the study, divided into two groups based on the presence or absence of pleural effusion (PE). Of these, 100 children had MPP with pleural effusion, while 100 children had MPP without pleural effusion. In the MPP without pleural effusion group, the male-to-female ratio was 53:47, with a mean age of 5.10±2.59 years. In the MPP with pleural effusion group, the male-to-female ratio was 63:37, and the mean age was 6.35±2.51 years.

Inclusion Criteria: The study included children aged 1 to 14 years who met the diagnostic criteria for Mycoplasma pneumoniae pneumonia based on clinical symptoms, laboratory tests, and chest X-ray results. The diagnosis was confirmed through elevated Mycoplasma pneumoniae antibody titers or PCR positivity. Additionally, only children with complete medical records and detailed clinical and laboratory data were included, and informed consent was obtained from parents or guardians.

Exclusion Criteria: Children with evidence of other bacterial or viral pneumonia, severe extrapulmonary infections (e.g., sepsis, meningitis, pericarditis), underlying medical conditions (such as immune deficiencies, chronic lung diseases, heart diseases), or those who had received antibiotics within two weeks prior to the study were excluded. Children with incomplete data or who were ineligible for follow-up for other reasons were also excluded.

### Methods

This retrospective analysis compared the clinical manifestations, laboratory results, and therapeutic outcomes of children with Mycoplasma pneumoniae pneumonia complicated by pleural effusion and those with uncomplicated MPP. The study aimed to identify potential predictors and evaluate the effects of different treatment approaches.

### Data collection

The study collected data on children diagnosed with Mycoplasma pneumoniae pneumonia in the pediatric ward between January 2021 and December 2023. Information collected included age, gender, onset of symptoms, specific clinical manifestations, medication use, duration of fever during hospitalisation, and the duration of antibiotic administration.

### Physical examination

Upon admission, each child underwent a comprehensive physical examination, which included assessments of mental status, respiratory function, chest wall movement, and auscultation and percussion of the lungs.

### Blood tests, imaging, and sputum culture

For each child, 5 mL of fasting venous blood was collected. Complete blood count (CBC) was analysed using an automatic haematology analyser (Sysmex XN-1000, Sysmex Corporation, Kobe, Japan). C-reactive protein (CRP) levels were measured by chemiluminescent immunoassay (Siemens ADVIA Centaur, Siemens Healthcare Diagnostics, Tarrytown, NY, USA), and immunoglobulin (IgG, IgM, and IgA) levels were assessed using a visible immunoturbidimetric method with a spectrophotometer (Beckman Coulter IMMAGE 800, Beckman Coulter, Brea, CA, USA). Imaging assessments were conducted using chest X-rays and CT scans, with two experienced physicians reviewing and agreeing upon the results. Sputum samples were collected using sterile disposable sputum aspirators, and the samples were cultured on AGAR or chocolate media in a 5% CO2 incubator. Bacterial growth was monitored, and an automatic microbial analyser was used for identification after 24 hours.

### Disease course, staging, and treatment plan

Treatment strategies for the children included antimicrobial therapy, supportive care (e.g., oxygen therapy and nutritional support), and, if necessary, interventions such as pleural effusion drainage. The study also documented the treatment methods employed, any changes during treatment, the length of hospital stay, and follow-up care provided to the children.

### Observation indicators

The primary outcome measures in this study included clinical manifestations, laboratory results, imaging findings, and treatment responses. All children underwent blood tests (CBC, CRP, and immunoglobulins) and imaging evaluations (chest radiographs and chest CT scans) to assess lung involvement and the presence of pleural effusion. For treatment response, information on the methods of treatment, hospital stay duration, and clinical progress during follow-up were recorded.

### Statistical analysis

Descriptive statistics were applied to summarise the data, with means and standard deviations calculated for continuous variables. The independent sample t-test or Mann-Whitney U test was used to compare differences between groups based on data distribution. Categorical variables were analysed using the Chi-square test or Fisher’s exact test. Logistic regression models were used to assess potential risk factors and their association with the presence of pleural effusion, and odds ratios (ORs) with 95% confidence intervals (CIs) were calculated. All statistical tests were two-tailed, with a significance level of P < 0.05. Data analysis was performed using the SPSS statistical software (Version 25.0, SPSS Inc., Chicago, IL, USA).

## Results

### Baseline characteristics and clinical manifestations

There was no statistically significant difference in the male-to-female ratio between the MPP group with pleural effusion and the MPP group without pleural effusion (P>0.05). However, the children in the pleural effusion group were older than those in the non-pleural effusion group. Additionally, the average duration of hospitalisation was longer for the children with pleural effusion compared to those without it. Laboratory results revealed that the white blood cell count, CRP levels, and neutrophil percentage (N%) were significantly higher in the pleural effusion group than in the non-pleural effusion group (P<0.05). The number of children with pleural effusion was considerably lower in the autumn season compared to those without pleural effusion (P<0.001). No significant difference was observed in terms of consolidation between the two groups (P>0.05), as shown in Table 1.

**Table 1:** Comparison of baseline features and clinical findings.

	MPP without pleural<br>effusion group<br>(n=100)	MPP with pleural<br>effusion group<br>(n=100)	t/χ^2^	P
Gender (male/female) [n (%)]	53 (53%)/47 (47%)	63 (63%)/37 (37%)	2.053	0.152
Age (years)	5.10±2.59	6.35±2.51	-3.466	0.001
Length of hospital stay (days)	5.98±2.14	7.26±3.04	-3.443	0.001
White blood cells (×10^9^/L)	8.59±1.36	9.37±3.58	-2.037	0.043
CRP (mg/L)	9.90±7.01	30.22±24.01	-8.124	<0.001
N %	56.99±11.22	66.01±11.90	-5.515	<0.001
Seasonal distribution				
Spring [n (%)]	14 (14%)	21 (21%)	2.512	0.113
Summer [n (%)]	16 (16%)	25 (25%)	3.182	0.075
Autumn [n (%)]	42 (42%)	17 (17%)	8.947	<0.001
Winter [n (%)]	28 (28%)	37 (37%)	1.854	0.174
Condensing (positive/negative) [n (%)]	34 (34%)/66 (66%)	36 (36%)/64 (64%)	0.022	0.882

### Imaging and laboratory results

Imaging findings indicated that lung lesions were more commonly found on the left side in the pleural effusion group, whereas bilateral lesions were more prevalent in the group without pleural effusion (P<0.001). There were no significant differences between the two groups in terms of bronchoscopy and sputum culture results (P>0.05). Laboratory analysis revealed significant differences in the levels of immunoglobulin M (IgM) and immunoglobulin A (IgA) between the groups. The pleural effusion group had higher IgM levels, while the non-pleural effusion group had higher IgA levels. However, immunoglobulin G levels did not show a significant difference between the two groups (P>0.05), as presented in Table 2.

**Table 2:** Imaging and laboratory results.

	MPP without pleural<br>effusion group<br>(n=100)	MPP with pleural<br>effusion group<br>(n=100)	t/χ^2^	P
Distribution of pulmonary lesions<br>[n (%)] – left side	19 (19%)	40 (40%)	6.237	0.012
Distribution of pulmonary lesions<br>Imaging and laboratory results n (%)]<br>– right side	35 (35%)	48 (48%)	2.840	0.092
Distribution of pulmonary lesions<br>[n (%)] – both sides	46 (46%)	12 (12%)	17.005	<0.001
Bronchoscopy – Congestion<br>[n (%)]	32 (89%)	36 (73%)	2.751	0.097
Bronchoscopy mucus plug [n (%)]	4 (11%)	13 (27%)	3.448	0.063
Sputum culture Streptococcus [n (%)]	5 (71%)	4 (80%)	3.141	0.076
Phlegm culture – Catalamolla [n (%)]	2 (29%)	1 (20%)	1.258	0.262
IgG (mg/dL)	958.46±233.52	986.060±313.741	-0.706	0.481
IgM (mg/dL)	130.48±77.65	167.390±85.684	-3.192	0.002
IgA (mg/dL)	195.51±79.93	164.497±87.218	2.621	0.009

### Drug treatment plan

Regarding medication regimens, intravenous glucocorticoids were administered more frequently in the MPP with pleural effusion group compared to the MPP without pleural effusion group (P<0.05). However, there was no significant difference between the two groups in the use of other treatments, including macrolide antibiotics alone, combination therapy with macrolides and -lactam drugs, and intravenous propyl spheres, as presented in Table 3.

**Table 3:** Post-admission medication regimen [n (%)].

	MPP without pleural<br>effusion group<br>(n=100)	MPP with pleural<br>effusion group<br>(n=100)	χ^2^	P
Macrolides were used alone	71 (71%)	59 (59%)	2.659	0.103
Combined treatment with<br>macrolides and beta-lactam drugs	27 (27%)	35 (35%)	1.145	0.285
Intravenous glucocorticoids	12 (12%)	31 (31%)	9.599	0.002
Venopropyl bulb	0 (0%)	3 (3%)	-	-

### Treatment response and clinical improvement

Regarding treatment response, patients in the pleural effusion group experienced a significantly longer duration of fever and antibiotic use during hospitalisation compared to those without pleural effusion (P<0.001), as shown in Table 4.

**Table 4:** Comparison of treatment response and clinical improvement.

	MPP without pleural<br>effusion group (n=100)	MPP with pleural<br>effusion group (n=100)	t	P
Duration of fever during<br>hospitalisation (days)	2.50±1.20	3.50±1.40	5.074	<0.001
Duration of antibiotic use<br>during hospitalisation (days)	7.00±2.00	9.00±2.50	6.400	<0.001

### Multivariate logistic regression analysis

**Figure 1:**
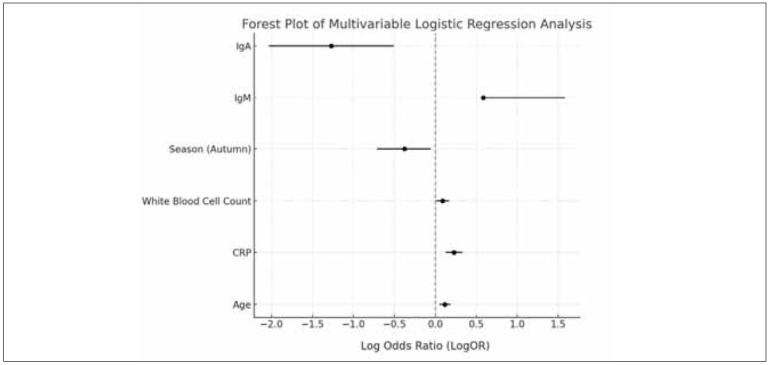
Multivariate logistic regression analysis.

Table 5 summarises the predictors of Mycoplasma pneumoniae pneumonia with pleural effusion in children based on multivariate logistic regression analysis. The findings indicate that the risk of pleural effusion increases by 12.2% with each additional year of age (OR=1.122, 95% CI: 1.051–1.203, P=0.001). Additionally, for every 10 mg/L increase in CRP, the risk rises by 25.5% (OR=1.255, 95% CI: 1.132–1.391, P<0.001), while a 1×10/L increase in white blood cell count is associated with a 9.2% higher risk (OR=1.092, 95% CI: 1.011–1.184, P=0.028). The likelihood of pleural effusion is lower in autumn compared to other seasons (OR=0.686, 95% CI: 0.491–0.942, P=0.021). Furthermore, higher levels of immunoglobulin M (IgM) significantly increase the risk (OR=1.795, 95% CI: 1.777–4.867, P=0.001), whereas elevated immunoglobulin A (IgA) levels significantly reduce it (OR=0.281, 95% CI: 0.131–0.602, P=0.001), as illustrated in Figure 1.

**Table 5:** Multivariate logistic regression analysis.

	value	SE	OR	95% CI	P
Age	0.113	0.031	1.122	1.051–1.203	0.001
CRP	0.221	0.054	1.255	1.132–1.391	<0.001
White blood cell count	0.091	0.046	1.092	1.011–1.184	0.028
Season (Autumn)	-0.391	0.162	0.686	0.491–0.942	0.021
IgM	0.318	0.281	1.795	1.777–4.867	0.001
IgA	0.267	0.288	0.281	0.131–0.602	0.001

## Discussion

The present study aimed to assess the prognostic value of serum C-reactive protein (CRP), Immunoglobulin M (IgM), and Immunoglobulin A (IgA) levels in children diagnosed with Mycoplasma pneumoniae pneumonia (MPP) complicated by pleural effusion. The findings provide valuable insights into the role of these biomarkers in predicting disease severity and complications, contributing to more effective clinical management of pediatric patients with MPP.

Pleural effusion is a significant complication of Mycoplasma pneumoniae pneumonia, often associated with increased disease severity, prolonged hospitalisation, and a greater need for intensive therapeutic interventions. The presence of pleural effusion suggests a more severe inflammatory response, which can be difficult to manage, especially in pediatric patients. Identifying biomarkers that predict the likelihood of pleural effusion development can enable earlier interventions, potentially improving clinical outcomes.

CRP is a well-established marker of inflammation and infection, and its levels were significantly higher in children with MPP complicated by pleural effusion compared to those without. This aligns with previous studies that have demonstrated a correlation between elevated CRP levels and the severity of pneumonia. The study found that for every 10 mg/L increase in CRP, the risk of developing pleural effusion rose significantly. This suggests that CRP could be a valuable prognostic marker for identifying children at higher risk of developing pleural effusion.

The significantly higher CRP levels in the pleural effusion group reflect an exaggerated inflammatory response, which may be due to bacterial invasion of the pleural cavity and increased production of proinflammatory cytokines. This finding highlights the potential utility of CRP in guiding clinical decisionmaking. For instance, children with markedly elevated CRP may require more aggressive treatment, including early administration of anti-inflammatory therapies such as corticosteroids.

IgM is the first immunoglobulin produced in response to an infection and is commonly used as a diagnostic marker for Mycoplasma pneumoniae infection. The study found that IgM levels were significantly higher in children with pleural effusion, with an increased risk of nearly 80% per unit increase in IgM. This suggests that IgM may serve as a prognostic marker, reflecting a more intense immune response that predisposes patients to complications.

Elevated IgM levels may indicate a higher bacterial load or a more severe immune reaction, leading to increased pulmonary inflammation and a greater likelihood of pleural involvement. These findings are consistent with previous research suggesting that a stronger IgM response is associated with severe M. pneumoniae infections. Clinically, this indicates that measuring IgM levels in children diagnosed with MPP could help identify those at risk for complications, allowing for closer monitoring and early intervention.

In contrast to IgM, IgA levels were significantly lower in the pleural effusion group, and higher IgA levels were associated with a decreased risk of pleural effusion. IgA is primarily involved in mucosal immunity, playing a crucial role in protecting the respiratory tract against pathogens. The reduced IgA levels in children with pleural effusion may suggest a diminished mucosal immune response, making them more susceptible to severe infection and complications.

The inverse relationship between IgA levels and the risk of pleural effusion could be due to its role in preventing bacterial adherence to respiratory epithelial cells and modulating inflammatory responses. This finding raises the possibility that children with lower IgA levels may have a weaker mucosal defence against M. pneumoniae, predisposing them to more severe disease manifestations. Further research is needed to explore whether interventions aimed at enhancing IgA-mediated immunity could reduce the risk of pleural effusion in MPP patients.

The results of this study have several important clinical implications. The identification of CRP, IgM, and IgA as significant prognostic markers allows for early risk stratification in children with MPP. Patients with high CRP and IgM levels, along with low IgA levels, may require more intensive monitoring and early intervention. Given that elevated CRP and IgM levels are associated with a higher risk of pleural effusion, early administration of anti-inflammatory and immunomodulatory therapies (e.g., corticosteroids) may help mitigate the inflammatory response and reduce complications. The study found that intravenous glucocorticoids were used more frequently in the pleural effusion group, suggesting a role for antiinflammatory treatment in managing severe cases.

Cao and colleagues evaluated the importance of monitoring mycoplasma pneumoniae-specific antibody IgM, CRP, and PCT levels in diagnosing mycoplasma pneumoniae pneumonia in children [13]. Their study showed that CRP and PCT levels were significantly higher in affected children compared to healthy controls. The combined detection of IgM, CRP, and PCT proved to be more accurate than individual tests, offering higher sensitivity, specificity, and predictive value. The authors concluded that this combination is a reliable diagnostic tool and helpful in assessing recovery in children with Mycoplasma pneumoniae pneumonia.

While macrolide antibiotics are the mainstay of M. pneumoniae treatment, children with high CRP and IgM levels may require prolonged or combination antibiotic therapy due to their increased risk of complications. The findings suggest that these biomarkers could be used to guide antibiotic duration and choice in clinical practice [14].

Serial measurement of CRP, IgM, and IgA levels during hospitalisation may provide valuable insights into disease progression. A rising CRP or IgM level could indicate worsening inflammation and a higher risk of pleural effusion, while stable or increasing IgA levels may suggest a better prognosis [15].

Despite its valuable findings, this study has some limitations. First, it was a retrospective analysis, which may introduce selection bias. Second, the study was conducted at a single institution, and its findings may not be generalisable to all populations. Third, while CRP, IgM, and IgA were found to be significant prognostic markers, other potential biomarkers (e.g., IL-6, TNF-α) were not examined [13].

Future research should focus on prospective, multicenter studies to validate these findings. Additionally, investigating the role of other inflammatory and immune markers in MPP with pleural effusion could further enhance our understanding of disease pathophysiology and improve risk prediction.

## Conclusion

This study provides compelling evidence that serum CRP, IgM, and IgA levels have significant prognostic value in children with Mycoplasma pneumoniae pneumonia complicated by pleural effusion. Elevated CRP and IgM levels were associated with a higher risk of pleural effusion, whereas higher IgA levels appeared to be protective. These findings highlight the potential role of these biomarkers in risk stratification, early diagnosis, and personalised treatment planning for pediatric patients with MPP. By integrating these markers into clinical practice, physicians can improve early detection of severe cases and optimise management strategies, ultimately leading to better patient outcomes.

## Dodatak

### Conflict of interest statement

All the authors declare that they have no conflict of interest in this work.
